# How to Support Parents of Infants and Young Children in Mental Health Care: A Narrative Review

**DOI:** 10.3389/fpsyg.2021.745800

**Published:** 2021-11-16

**Authors:** Hanna Stolper, Karin van Doesum, Majone Steketee

**Affiliations:** ^1^Department of Psychology Education and Child Studies, Erasmus University Rotterdam, Rotterdam, Netherlands; ^2^Department of Clinical Psychology, Radboud University, Nijmegen, Netherlands

**Keywords:** parental mental disorder, infants and early childhood, intergenerational transmission of psychopathology, targets of intervention, review

## Abstract

**Objective:** The aim of this narrative review is to gain insight into the appropriate intervention targets when parents of infants and young children suffer from psychopathology.

**Background:** Psychopathology in parents is a risk factor for maladaptive parenting and is strongly related to negative cascade effects on parent-child interactions and relations in the short and long term. Children in their first years of life are especially at risk. However, in adult mental health care, this knowledge is rarely translated into practice, which is a missed opportunity for prevention.

**Methods:** Electronic databases were searched for reviews and meta-analysis. In addition, sources were obtained via manual search, reference mining, expert opinion, and communications from conferences. In total, 56 papers, whereof 23 reviews and 12 meta-analyses were included.

**Results:** Findings regarding targets of intervention were identified in different interacting domains, namely the parental, family, child, and environmental domains as well as the developing parent-child relationship. A “one size fits all” intervention is not appropriate. A flexible, tailored, resource-oriented intervention program, multi-faceted in addressing all modifiable risk factors and using different methods (individual, dyadic, group), seems to provide the best results.

**Conclusion:** To address the risk factors in different domains, adult and child mental health care providers should work together in close collaboration to treat the whole family including mental disorders, relational, and contextual problems. A multi-agency approach that includes social services is needed.

## Introduction

Children of parents with a mental disorder are at increased risk for developing mental health problems during their lifetime. The degree of transmission of psychopathology from parent to child ranges from 41 to 77% for the whole diagnostic spectrum (Hosman et al., 2009).

The risk for the child appears to be greater during pregnancy and early life, because these phases are crucial for the development of the brain and building a secure attachment relationship that impacts the development of the young child (Agorastos et al., 2019; Aktar et al., 2019). Pregnancy, childbirth, and parenting are likely to be more challenging for parents with a mental disorder, and as a consequence may aggravate the psychopathology of the parent(s) (Barker et al., 2012; Falkov, 2012; Aktar et al., 2019). The transactional model (Sameroff, 2004) illustrates how the reciprocal nature of the parent-infant relationship over time affects both parent and child. For instance, if the parent is inadequately responsive to the infant due to their psychiatric symptoms, resulting in under-, over- or highly unpredictable stimulation, the infant will experience confusion and feel unsafe during interactions with the parent, which puts them at risk for developmental delays, insecure attachment styles, challenging behavior, and for the parent, less satisfying and more stressful parenthood. Hence, early childhood is an essential time window for the prevention and treatment of unfavorable parent-child interaction cascades. Therefore, in addition to treatment of the mental disorder of the parent, it is important to pay close attention to parenthood and the evolving parent-child relationship and to act as early in the child’s life as possible to repair negative parent-infant interaction patterns (Forman et al., 2007). However, it remains unclear what should be the targets and means of both parent and child in mental health care to reduce the risk of psychopathology during infancy and early childhood.

**FIGURE 1:**
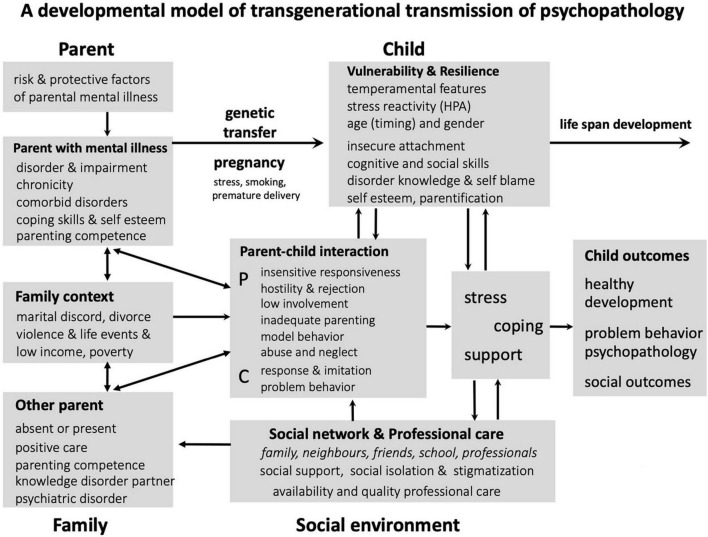
Transmission mechanisms and factors in families with parental mental disorders (Hosman et al., 2009).

The high degree of intergenerational transmission does not take place via a direct or simple pathway. Reupert et al. (2015) provide an overview of a number of different, mostly overlapping conceptual models that provide insight into the factors and mechanisms of transmission. The integrative model of Goodman and Gotlib (1999) for transmission of risk to children of depressed mothers is based on empirical data and clarifies the interrelated and interacting risk factors. Falkov (2012) developed the family model to promote transformation in mental health services to focus on the family rather than concentrating solely on the individual, with the aim to prevent the intergenerational transmission of psychopathology. Hosman et al. (2009) present a developmental model of intergenerational transmission of psychopathology with risk and protective factors within four different domains (parent, child, family, and environment) many of which are related to parental mental disorders. This model, as presented in Figure 1, is comprehensive in providing insight into which factors and mechanisms could be involved in the process of transmission.

However, there is not yet an all-encompassing framework that predicts and clarifies the developmental pathway by which parental mental disorder affects children, for this process is influenced by the presence and accumulation of interacting risk and protective factors (Maybery et al., 2015; Reupert and Maybery, 2016).

The objectives of this paper are, first, to identify important and effective intervention targets when parenting of infants and young children is included in the treatment of parents with psychopathology, in order to break through the cycle of intergenerational transmission of psychopathology, and second, to determine which among all the mentioned targets should have higher priority in order to achieve the goal of reducing the risk of transmission of psychopathology. In line with the purpose of this paper, we take a developmental-transactional perspective (Sameroff, 2004; Sroufe et al., 2009), which means that psychopathology develops through an interaction between person and environment and evolves over time. Although most research focuses on parents with a specific classification according to the Diagnostic and Statistical Manual of Mental Disorders-5 (DSM-5; American Psychiatric Association [APA], 2013), in clinical practice there is wide variety in the phenomenology of mental disorders (Allsopp et al., 2019). Therefore, we will not focus on a specific classification to avoid the false impression that one is dealing with a homogeneous group that can be treated in a uniform way. We chose a narrative review because we wanted to include a wide range of data from a diverse body of literature, and many sources do not lend themselves to a quantitative pooling of results. The focus for this review will be on parents with serious psychopathology, predominantly of a chronic and complex type, that would be encountered in mental health care settings.

In this narrative review, we will present findings about intervention targets in mental health care to break the cycle of transmission of mental disorders and adverse outcomes. This covers intervention targets within different interacting domains, namely the parental, family, child, and environmental domains as well as the developing parent-child relationship.

## Materials and Methods

Electronic databases (PsycINFO and Web of Science) were searched for reviews in the period 2009–2021 using the following search terms: intergenerational transmission of psychopathology, OR parents with a mental illness, OR parental psychiatric disorder, AND/OR risk and protective factors, AND infants, OR infancy, AND interventions, AND reviews, AND Children of Parents with a Mental Illness (COPMI), AND Children of Mentally Ill Parents at Risk Evaluation (COMPARE). Because of the broad topic of this literature review and the variety of scientific fields that are involved (e.g., parental psychiatry, developmental psychopathology, infant mental health, attachment, resilience science, trauma and toxic stress, genetics and epigenetics, neuroscience and neuroendocrinology), we first searched for reviews and 916 hits of research papers and reviews were found. After reading the title of all, 117 were selected as relevant to the topic and after reading the abstract 33 for close reading, whereof six papers were included in the review. The inclusion criteria were: (1) risk and protective factors for child’s psychopathology and adverse outcome related to parental mental disorders; (2) the period of infancy or early childhood; (3) young child, and family resilience; (4) interventions; (5) review; (6) meta analytic study; (7) English language; (8) peer reviewed. Exclusion criteria were: (1) child’s cognitive development or school achievement related to parental mental disorder; (2) risk factors to specific child mental health disorders, such as ADHD, autism spectrum disorders, not specific related to parental mental disorders; (3) interventions preventing parental mental disorder not related to child outcomes; (4) interventions whereof the period of early childhood was not a substantial part; (5) interventions related to physical health of the young child (medical, nutrition). However, crucial risk and protective factors in relation to intervention targets to prevent the transmission of psychopathology during infancy and early childhood according to the model of Hosman et al. (2009) were missing (e.g., attachment, resilience, adverse child experiences). We conducted additional hand searching and reference mining, alongside obtaining information from other sources such as communications at conferences and correspondence with experts. This resulted in 17 new reviews, so a total of 23 reviews were included. In addition, 12 meta-analytic studies were included. We could not find any reviews or meta-analyses on the particular impact and intervention targets in the presence of cumulative risk factors, poly victimization, and how to promote resilience during infancy and early childhood. On these particular topics we searched for papers in the same manner as described above, using the same inclusion and exclusion criteria and 21 papers about intervention targets have been added. In summary, this review on appropriate intervention targets when parents of young children suffer with psychopathology is based on 56 publications.

## Results

Because no reviews have been found about different intervention targets related to risk and protective factors for parents with psychopathology and their young children in mental health care, the focus in this section is to present an overview of the risk and protective factors that are specific to the phase of early childhood. In particular, the modifiable factors for the different domains (parental, parent-child relationship, family, child, and environmental) are subsequently reviewed in relation to intervention targets. We finally end by summarizing risk factors, and the targets of intervention which aim to modify the risk factors into protective factors.

### Risk and Protective Factors

Interaction mechanisms among risk and protective factors in the four different domains, as presented in Figure 1, highly impact child’s development and mental health outcome. Therefore, all risk factors suggest directions in treatment of parents and their young child to prevent the risk of intergenerational transmission of psychopathology (Christiansen et al., 2019). Risk factors related to the parental, family, child, and environmental domains, with references to the literature, are presented in Table 1.

**TABLE 1:** Risk factors during pregnancy and early childhood in different domains related to parental mental disorder.

Parental domain	Family domain	Child domain	Environmental domain
Genetic transmission^1^	Early parenthood^16,17^	Difficult temperament^4,6,2,3^	Low socio- economic status^16,31,32^
Maternal anxiety and stress in pregnancy^2,3^	Single parenthood^16,17^	Mental health disorders^23^	Poverty^4,18,17,33^
Severity, chronicity or early onset of the disorder^4,5,6^	Child abuse (physical, emotional, sexual)^4,18,19,20^	Genetic vulnerability to environmental influences^24^	Housing problems^18,32^
Comorbidity (e.g., substance abuse)^4,6^	Child neglect (physical, emotional)^18,19,20^	Effects of early life stress and trauma/ACEs^25,26^	Social isolation^18,32^
Unresolved (childhood) trauma^7,8,9^	Unpredictable or lack of daily routines^11,21^	Insecure and disorganized attachment^4,6,10,27,28,29,30^	Belonging to a minority group^34^, perceived discrimination^32^
Absence of treatment^6,10^	Mental disorder/addiction other parent/family member^22,6,19^		Low quality of neighborhood^18,32^
Problematic parenting^4,11,12,13,14^	Interparental conflict/violence^4,16,18,19,12,20^		Absence or low quality of emotional and practical support network^16,32^
Frightened, frightening disrupted parental behavior^8^	Absent of both biological parents^19^		Absence or low quality of adult and child professional care^6^
Impaired and distorted parental mentalization^15^	Criminal trouble/imprisoning family member^3,12,18^		No possibilities for alternative care^18^
	Low level of education^16,17^		

*^1^Kendler and Prescott, 2007; ^2^Korja et al., 2017; ^3^Van den Bergh et al., 2017; ^4^Beardslee et al., 2011; ^5^Carter et al., 2001; ^6^Hosman et al., 2009; ^7^Christie et al., 2019; ^8^Madigan et al., 2006; ^9^Suardi et al., 2017; ^10^Aktar et al., 2019; ^11^Falkov, 2012; ^12^Hughes and Cossar, 2016; ^13^Hugill et al., 2017; ^14^Laulik et al., 2013; ^15^Sharp and Fonagy, 2008; ^16^Barker et al., 2012; ^17^Evans et al., 2013; ^18^Brockington et al., 2011; ^19^Felitti et al., 1998; ^20^Kessler et al., 2010; ^21^Stepp et al., 2012; ^22^Bijl et al., 2002; ^23^Slade, 2009; ^24^Bakermans-Kranenburg and van IJzendoorn, 2007; ^25^Agorastos et al., 2019; ^26^Chu and Lieberman, 2010; ^27^Fearon et al., 2010; ^28^Granqvist et al., 2017; ^29^Sroufe et al., 2009; ^30^Van IJzendoorn et al., 1999; ^31^Stein et al., 2014; ^32^Silva et al., 2016; ^33^Lund and Cois, 2018; ^34^Cyr et al., 2010.*

Protective factors which can play a protective role in the transfer of psychopathology in the different domains are presented in Table 2.

**TABLE 2:** General protective factors in early childhood in different domains, which may have a buffering effect on families where a parent has a mental disorder.

Parental domain	Family domain	Child domain	Environmental domain
Physical health^1^	Presence of a well-functioning other (step-) parent^7,8^	Physical health^1^	Stable and adequate income^1,6,15^
Self- and parental efficacy^1,2,3^	Warm, cohesive family interaction^1^	Easy temperament^1,10,7^	Adequate housing^1^
Good emotion and stress regulation^1,4^	Marital stability, support and satisfaction^1,3,4,6,9^	Insusceptible for environmental influences^11^	Safe neighborhood^1,15,16^
Effective coping skills^1,2,4^	Small family (<4 children)^1^	Secure attachment^1,2,4,12,13,14^	Social support (emotional/practical) ^1,6,7,16,17^
Internal locus of control^1^	Moderate or high level of education^1,6^		Involvement in the community^1,6,15^
Positive belief systems^1,2,3^	Supportive and stimulating parent-child interaction^1,2,4^		Access to good quality childcare and school^1,4,6^
Appropriate parental mentalization^5^/secure attachment^6^			Access to good quality (mental) health care^1,6,7,16^

*^1^Benzies and Mychasiuk, 2009; ^2^Doty et al., 2017; *^3^*Korja et al., 2017; ^4^Masten, 2018; ^5^Slade et al., 2005; ^6^Tharner et al., 2012; ^7^Hosman et al., 2009; ^8^Stein et al., 2014; ^9^McEwen, 2003; ^10^Beardslee et al., 2011; ^11^Bakermans-Kranenburg and van IJzendoorn, 2007; ^12^Seifer, 2003; ^13^McGoron et al., 2012; ^14^Schechter and Willheim, 2009; ^15^Silva et al., 2016; ^16^Brockington et al., 2011; ^17^Falkov, 2012.*

An epidemiologic study by Kessler et al. (2010) showed that childhood adversities often co-occur. Several studies indicate a dose-dependent response relation of childhood adversities to mental health outcomes, which means that the number of traumas and adversities is a significant predictor of mental health disorders (Barker et al., 2012; Evans et al., 2013). A longitudinal study (Barker et al., 2012) investigated the impact of the exposure of infants from birth to 2 years old to several risk factors and the impact of maternal depression on child psychopathology at 7 years. They found evidence that a substantial proportion (37–41%) of the association between risk factors and maternal depression was explained by increased risk factor exposure. Beside exposure to maternal depression, each additional risk factor increases the odds at least 20% for child psychopathology.

### Targets of Intervention in Different Domains

The following section will describe intervention targets in the domain of the parent, the family, the child, and the environment, and the early parent-child relationship as a crucial transmission mechanism, all of which are considered to be important in limiting the risk of transmission of psychopathology from parent to child.

#### Parental Domain

##### Genetic transmission and epigenetic regulation

The risk of *transmission of specific genes* from parent to child which increases the probability of psychopathology in the child (Kendler and Prescott, 2007), is not a modifiable factor. This significant risk justifies categorization as a high-risk group that needs preventive interventions. Epigenetic changes in transcription of genes have been shown in interaction with the environment, especially in early development and following adverse experiences. *Maternal anxiety and stress during pregnancy* are major risk factors for later negative child difficulties (Van den Bergh et al., 2017). Exposure to early life stress and childhood trauma, possibly arising from parental psychopathology, further enhance the risk of epigenetic programming which over time may lead to stress-related disorders (Agorastos et al., 2019). The quality of the parental environment is an important factor that can be modified and therefore it is a crucial target of intervention.

##### Parenting in relation to psychopathology

Much is known about how parenting can be affected by a mental disorder. Most studies address concerns related to depression in mothers and its influence on their caregiving such as unresponsiveness, intrusiveness, hostility, or high expressed emotions (Beardslee et al., 2011). There is also evidence of problematic parenting by parents with a personality disorder, for instance switches between hostile control and withdrawn behaviors as well switches between intrusiveness and coldness (Stepp et al., 2012; Laulik et al., 2013). An association has been found between PTSD and impaired parenting and less than optimal parent-child relationships (Christie et al., 2019). A systematic review of the relationship between *maternal childhood trauma* (emotionally abusive or neglectful experiences) and parenting found tentative support for a range of adverse parenting outcomes, including increased parenting stress and a higher risk of maltreatment, lower empathy, and greater psychological control (Hughes and Cossar, 2016). Hugill et al. (2017) found support for an indirect pathway from childhood sexual abuse of parents through parental depression to parenting stress. Notwithstanding the above mentioned risks, parenting can also be a positive resource for parents with mental disorders, as it may be a source of structure and stability and act as a motivator to manage their symptoms in a better way (Schrank et al., 2015).

##### Problematic parenting behavior and distorted parental mentalization

Parenting can be a key moderating or mediating factor in transmission of mental disorders from parent to child and therefore should be an important target of intervention (Brockington et al., 2011; Stein et al., 2014). Specific *problematic parental behavior* related to mental health problems includes overinvolvement (intrusiveness and/or overprotection), underinvolvement and neglect (unresponsiveness to physical and emotional needs), negative involvement (hostility and irritability), inconsistent involvement (inconsistency in providing daily structure and predictability), inadequate discipline and control (harsh discipline and criticism), role reversal (parent seeks comfort from child), developmentally inappropriate expectations, and lack of modeling (Falkov, 2012).

Parental mentalization or parental reflective functioning (PRF) refers to the parent’s capacity to understand their own as well as their child’s internal states. It allows them to regulate and comfort their child in an appropriate way and for that reason plays a vital role in the development of attachment and the child’s self-regulation and capacity to mentalize (Slade et al., 2005). *Distorted parental mentalization* is associated with disorganized attachment and development of psychopathology in their children (Sharp and Fonagy, 2008). Low reflective function (RF) has been found among adults with different types of mental disorders (Katznelson, 2014).

##### The nature of the parental mental disorder

The chance that the child will develop a disorder is not strongly dependent on the specific disorder of the parent (Bijl et al., 2002). *Severity, chronicity, and degree of comorbidity of parental psychopathology* has been shown to contribute more to parental behavior than specific disorders (Carter et al., 2001) and to the likelihood of transmission (Beardslee et al., 2011). In this context, a*bsence of treatment* is an obvious risk (Hosman et al., 2009; Beardslee et al., 2011). *Early onset* (before age 30) of a disorder is an additional risk, because of the higher likelihood of adverse social, educational and work circumstances found in young mothers (Hosman et al., 2009).

Allsopp et al. (2019) demonstrate that most DSM-5 classifications are associated with adversities or trauma in childhood. Acknowledging the possible role of trauma or adversity in the unfolding of psychopathology, interventions should also address past traumatic experiences. This is crucial because the presence of *unresolved childhood trauma* in parents is known to be an important threat to their parenting, for example, in *frightened, frightening, and disrupted behavior*, and the early parent-child relationship (Madigan et al., 2006; Suardi et al., 2017). Indeed, parents’ post-traumatic stress symptoms may impair self-regulation and the parent’s ability to regulate the young child, who depends greatly on interactive co-regulation with the caregiver (Chu and Lieberman, 2010; Suardi et al., 2017). The child’s behavior or distress itself can provoke and revive the post-traumatic stress symptoms caused by unresolved trauma experienced by the parent. In this process, parent and child may be caught in an unintended repeating traumatic interaction circle. Therefore, screening and treatment for unresolved parental childhood trauma will benefit the parent, the child, and their relationship (Chu and Lieberman, 2010; Suardi et al., 2017).

Although treatment of parents’ mental disorders and trauma can play a role in the prevention of mental disorders in their children, interventions targeting the parent-infant relationship have been shown to produce larger overall effects in improvement of this dyad than individual interventions targeting mothers only (Kersten-Alvarez et al., 2011; Tsivos et al., 2015; Thanhäuser et al., 2017; Aktar et al., 2019). Evidence suggests that explicit attention has to be paid to the parent-child relationship in order to improve the emergence of undesirable patterns, such as a lack of parents’ responsiveness to the child (Carter et al., 2001; Nylen et al., 2006; Forman et al., 2007; Schechter and Willheim, 2009; Barlow et al., 2010; Barker et al., 2012; Barnes and Theule, 2019).

##### Summary

Parental mental disorders may be a serious threat to the quality of parenting. Parenting is a key moderating and mediating factor in the process of transmission of mental disorders. The capacity of the parent to understand their child’s emotion and behavior and to help their child’s regulation in appropriate ways depend on parental mentalization. Parental behavior and parental mentalization as highly influential factors are strongly related and therefore important targets for intervention.

The severity, chronicity, comorbidity, and early onset of parental mental disorder and unresolved parental childhood trauma increase the likelihood of intergenerational transmission of psychopathology. Treatment of the disorder and unresolved childhood trauma of the parent(s) should be accompanied by explicit attention to the developing parent-child relationship in order to prevent the emergence or continuation of undesirable parent-child interaction patterns.

#### The Early Parent-Child Relationship

The parent-child relationship is generally the most proximal and influential relational system for the child. Preliminary support in clinical samples suggest a link between higher levels of *insecure attachment* in infants and parental behavior related to mental disorders, but further research is needed (Aktar et al., 2019; Barnes and Theule, 2019). Seifer (2003) argued that fostering resilience of very young children of a parent with a mental disorder on the individual level is complicated, as they have not developed the functions and skills needed to develop resilience such as verbal skills and high-level cognitive functioning. Furthermore, they function in limited social contexts and miss independent access to various social communities that are assumed to be a protective factor for older youth. The focus of the resilience process needs to be the parent-child system (e.g., attachment) as an interim outcome because if there are unresolved disturbances in this relationship it will increase the risk of adverse child outcomes (Seifer, 2003). A positive change in that relationship will have positive spillover effects over time on other domains with long-term benefits to the parent (parental efficacy, positive emotions) and child (cognitive, emotional, and social functioning) (Doty et al., 2017).

##### Enhancing secure attachment as a protective factor

Secure attachment can be seen as an interim outcome and a buffering protective factor against the development of psychopathology (Seifer, 2003; Schechter and Willheim, 2009; McGoron et al., 2012). Parental sensitivity to children’s cues is associated with and currently seen as an important—but not the only—predictor of secure attachment (Bakermans-Kranenburg et al., 2003), and for that reason is a key target in many attachment-based interventions. Parental sensitivity refers to the ability to accurately perceive and interpret the infant’s signals and communications and respond appropriately. However, a recent meta-analysis (Zeegers et al., 2017) of parental mentalization and sensitivity as predictors of infant-parent attachment highlights a direct effect of parental mentalization on infant-parent attachment independent of parental sensitivity, as well as an indirect impact on attachment via its effect on sensitivity. Therefore, parental mentalization as well as parental sensitivity are important targets for interventions to enhance secure attachment. This implies helping parents to think about behavior as an expression of the intentions and internal mental states of their child. It also necessitates changing the parental representation of the child, with the result that the parent is able to see the child as having an inner life separate from their own (Slade et al., 2005).

##### Reducing the risk of insecure and disorganized attachment

*Insecure and especially disorganized attachment* in young children has been associated with later mental health outcomes such as problematic stress management, externalizing and dissociative behavior, and borderline personality disorder (Van IJzendoorn et al., 1999; Sroufe et al., 2009; Fearon et al., 2010). Disorganized attachment is the opposite of organized attachment, and entails the child becoming stuck in an unresolvable conflict between being afraid of the parent and at the same time needing comfort from the parent. This conflict, described as “fright without solution,” is observable in a breakdown of attachment strategies in a stressful situation in the presence of the parent. For instance, it may involve contradictory behavior such as seeking proximity to and turning away from the parent, and stereotyped behavior such as extended rocking. On a biobehavioral level there is a measurable increased level of cortisol (Spangler and Grossmann, 1993).

There are different pathways to disorganized attachment, and one is directly related to parental psychopathology. An association has been found between subtly frightened, frightening, and disrupted maternal behavior and disorganized attachment in infancy (Madigan et al., 2006). Due to unresolved parental trauma and loss, parents themselves may become sources of chronic stress for their infants because of an ongoing state of fear, dissociative behavior, limited availability and responsiveness, and restrictive and overprotective behavior (Chu and Lieberman, 2010; Suardi et al., 2017).

Given the association between disorganized attachment and later mental health outcomes, preventive interventions targeting attachment relationships in infancy are needed. A recent meta-analysis (Facompré et al., 2018) demonstrates that interventions targeting disorganized attachment have been generally effective. The interventions in the studies focused on enhancing parental sensitivity to the infant’s cues, on modifying the parental representations of the child in relation of the caregiver’s own attachment history, and on the effect of practical support and education on child development. The findings show no significant differences among the intervention foci. Another meta-analysis of interventions that aim to decrease or to prevent disorganized attachment in early childhood shows a reduction in disorganized attachment, with the majority of the interventions focusing on maternal sensitivity (Wright et al., 2017). On the other hand, Benoit et al. (2001) argue that interventions should target disorganizing interactions between caregivers and their children. Instead of blaming parents for their children’s disorganized attachment, Granqvist et al. (2017) emphasize that caregivers need help to learn to follow the child’s lead, avoid alarming behavior and provide nurturance, make sense of traumatic experiences, break social isolation, and learn strategies to remain with the child in the moment rather than become lost in memories.

##### Summary

The way parents interact with their infants is essential for building a secure attachment relationship during the first year of life, and is associated with healthy social and emotional development outcomes. Secure attachment can be seen as an intermediate outcome and a buffering protective factor against the development of psychopathology. Given the association between disorganized attachment and later mental health outcomes, preventive and curative interventions targeting attachment relationships in infancy and early childhood is a desirable response. Intervention should target enhancing parental mentalization and sensitivity, reducing disorganizing interactions between caregivers and their children, promoting practical support, and education about child development.

#### Family Domain

The parent and the parent-infant relationship usually function in the broader context of family life. Family factors, such as the functioning of the other (co-)parent, quality of the partner relationship, and family functioning are strong predictors of mental health outcomes. *Low level of education*, e*arly parenthood, and single parenthood* are not modifiable risk factors (Barker et al., 2012; Evans et al., 2013), although attention can be paid to diminish the social consequences for instance by social work interventions.

##### The other (co-)parent

Children whose *parents both have one or more mental disorders* have an increased risk of developing a disorder themselves (66%) compared to children whose one parent has a mental disorder (51%), and almost twice as high compared to children with parents without a mental disorder (35%) (Bijl et al., 2002). Healthy functioning of the other parent is mentioned as an important protective factor because it might have a buffering effect on the impact of the parent with a mental disorder on the child, and for this reason the (co-)parent should be routinely involved in the assessment phase of treatment (Stein et al., 2014). Improved child outcomes have been found when fathers are involved in family focused interventions (Harold and Sellers, 2018).

##### Quality of the partner relationship

This is mentioned as an important protective or risk factor because of its direct impact on the parental mental disorder and indirect impact on the child’s outcome. Social support through a loving and caring relationship with a partner appears to have an ameliorative effect on psychopathology caused by early life adversity (McEwen, 2003).

*Interparental conflicts* predict children’s problematic functioning even after controlling for other family and ecological characteristics (Cummings and Davies, 2002). A recent study found that the association between fetal exposure to parental mood disorder and children’s internalizing problems at 24 months is mediated by poor postnatal quality of the couple’s relationship (Hughes et al., 2019). This study recommends clinical interventions to improve couple relations during pregnancy to benefit the child’s later outcomes. Korja et al. (2017) mentioned marital support and satisfaction as moderators that may attenuate the negative impact of prenatal exposure to maternal stress, depression, and anxiety.

Given the high prevalence of *interparental violence* against women with perinatal mental disorders (depression, anxiety, and PTSD), mental health services should identify and respond to interparental violence against women they treat (Howard et al., 2013). In the presence of interparental conflicts, interventions that target these conflicts at the level of the interparental relationship may benefit the child’s psychopathological outcomes in the long term significantly more than interventions that target the parent-child relationship (Harold and Sellers, 2018). Therefore, the interparental relationship should get priority.

##### Quality of family functioning

Benzies and Mychasiuk (2009) mentioned warm interaction and family cohesion as one of the most significant protective factors for families with a parent who has a mental disorder. It provides family members a safe haven for development and is known as a buffering factor which protect children from the negative consequences related to low-income. Fostering protective factors in the family is a strength-based intervention which promote family resilience to cope with adverse events.

*Unpredictable or lack of daily routines* is a risk factor (Falkov, 2012), often associated with the presence of a borderline personality disorder at the parent, which should be prioritized over attachment issues (Stepp et al., 2012). Kessler et al. (2010) analyzed data from 21 countries and found that childhood adversities associated with maladaptive family functioning such as parental mental disorder, *interparental violence*, *criminal behavior*, *neglect*, and *physical and sexual abuse* of the child were the strongest predictors of mental disorders over the course of the child’s lifetime. Child maltreatment is associated with disorganized attachment and adverse outcomes (Cyr et al., 2010). The prevalence of child maltreatment is elevated in the presence of a parental mental disorder, even more so when both parents are affected (Chang et al., 2018). In a review regarding the impact of traumatic stress from birth to age 5, Chu and Lieberman (2010) argued that the prevalence of trauma in early childhood is highly underestimated and seldom investigated by researchers. Childhood trauma and stress has been estimated to account for 45% of the variance of psychopathology beginning in childhood and 26–32% in adulthood (Tyrka et al., 2013). Finkelhor et al. (2007) highlight the role of poly-victimization, a phenomenon neglected by researchers and practitioners. If a child was exposed to one kind of trauma or adverse experience it is more likely that they will have been exposed to additional traumas. For this reason, they argued that professionals need to assess for a broader range of traumas and early interventions. The impact of traumatic stress depends on the quality of the parent-child relationship, because for the infant the attachment relationship is an important resource for regulating emotions and stress (Chu and Lieberman, 2010; Shonkoff et al., 2012). The child’s traumatic experiences should be treated in the context of the caregiver-child relationship (Chu and Lieberman, 2010).

A parent with psychopathology is one of the risk factors in the Adverse Child Experiences (ACE) Study (Felitti et al., 1998), a huge epidemiological study in the United States which has been replicated several times. It shows how adverse childhood experiences or traumas from infancy to 18 years of age can lead to medical disease and psychopathology. The effects of ACE start in early childhood and can be long-lasting (Felitti et al., 1998). The 10 empirically selected ACE categories, mostly related to family functioning, are *abuse (emotional, physical, sexual)*, *neglect (physical, emotional)*, and household dysfunction *(parental violence, household member was addicted, imprisoned, mentally ill or in psychiatric hospital, not raised by both biological parents)* (Liming and Grube, 2018). An individual ACE score is calculated by counting the number of categories experienced in childhood. The ACE Study found that the impact of the different ACE categories is more or less the same, and with an ACE of four or more, the risk of adverse outcomes is significantly increased. Young children age 0–6 with three or more ACEs were significantly more likely to exhibit behavioral problems (e.g., aggression, attention problems), mental health problems (e.g., anxiety), and overall problems compared with children with no ACEs (Liming and Grube, 2018).

##### Summary

Family factors can act as strong protective or risk factors for intergenerational transmission. Assessment of family factors is necessary and should include the role and mental health of the other parent, the quality of the couple relationship and family functioning, especially the presence of ACEs and trauma. Maladaptive family functioning is a strong risk factor. Interparental violence or marital conflict should get a higher priority for intervention than the parent-child relationship. Warm and supportive interactions between family members is a strong protective factor. To reduce the impact of ACEs and early childhood trauma, treatment should be in the context of the current attachment relationship.

#### Child Domain

##### Child vulnerabilities

Infant characteristics may function as a protective as well as a risk factor. Vulnerabilities may be independent of the parent’s psychopathology as well as result of exposure to risk factors related to parent’s psychopathology or an interaction of both. However, children’s vulnerabilities pose a risk for the developing parent-child relationship, for they challenge parenting and the mentalizing capacity of the parent (Sharp and Fonagy, 2008).

Differences in reactivity, activity and self-regulation are seen as features of temperament whereby a difficult temperament refers to negative affect or irritability, withdrawal in response to novelty, high intensity of emotions and irregularity of biological processes such as feeding and sleeping (Korja et al., 2017). An infant or young child with a difficult temperament demands much more effort from the caregiver in the interpersonal regulation compared to a child with an easy temperament. Therefore a *difficult infant temperament* is a frequently identified risk factor (Korja et al., 2017; Van den Bergh et al., 2017), and conversely an easy temperament is a protective factor in the presence of risk (Benzies and Mychasiuk, 2009; Beardslee et al., 2011).

In accordance with the fetal programming model, there is robust evidence that early negative environmental factors, such as maternal anxiety, depression or stress in pregnancy (Korja et al., 2017; Van den Bergh et al., 2017) constitute a major risk factor for negative outcomes for the child later in life due to the plasticity of biological systems of the fetus in adaptation to the environment. Korja et al. (2017) suggest that there is evidence of an association between higher prenatal stress and anxiety and elevated negative reactivity or poorer self-regulation, both features of a child’s temperament. Exposure to prenatal stress, possibly resulting in difficult childhood temperament, can increase the risk of later psychopathology, partly due to the impact of non-optimal parenting provoked by the child’s difficult temperament. Van den Bergh et al. (2017) found that maternal stress during pregnancy was related to increased risk for behavioral problems and a wide range of mental health problems in the offspring. Prenatal exposure to maternal anxiety or depression is associated with many aspects of brain functioning in offspring such as impulsivity and attention.

To prevent unborn children from suffering the negative effects of maternal stress during pregnancy, Van den Bergh et al. (2017) recommended that pregnant women should be protected from undue hardship and stress and advised to avoid preventable stressors. Korja et al. (2017) suggest maternal caregiving sensitivity, maternal self-efficacy, and marital support and satisfaction (see also Tharner et al., 2012) are moderators that may attenuate the negative impact of fetal programming. Based on these findings, they advise preventive approaches and active treatment to help mothers who are experiencing prenatal stress or anxiety and prevent their offspring from having long-term difficulties in self-regulation. To mitigate the risk of early transmission of psychopathology Aktar et al. (2019) suggest intensive treatment of prenatal and postnatal depression alongside with interventions targeting the mother-infant interactions.

Children with *mental health disorders* themselves according to the Diagnostic Classification of Mental Health and Developmental Disorders of Infancy and Early Childhood (DC:0-5^TM^) (Zero to Three, 2016), challenge the pleasure of and confidence in parenthood. For example, those on the autistic spectrum, who are born with fundamental problems in understanding social information and developing relationships with others, may be a challenge for their parents due to the lack of reciprocity. Parents of these children are in need of help connecting with their child (Slade, 2009).

As mentioned before *early life stress and trauma* leave their traces on child’s development (Agorastos et al., 2019) and may also contribute to an *insecure and disorganized attachment style*, which need treatment as discussed in the section on the early parent-child relationship (Granqvist et al., 2017).

##### Susceptibility to environmental influences

Bakermans-Kranenburg and van IJzendoorn (2007) highlight the role of *genetic factors in creating differences in susceptibility* to positive or negative environmental influences. If there is a high susceptibility to environmental influences, risk factors such as maltreatment or neglect will have a highly negative impact on child development, whereas a supportive environment will be of great benefit and contribute to the development of resilience. If there is a low susceptibility to environmental influences, there is less impact of negative child rearing experiences on children’s development, but there is also little benefit from treatment. These individual differences underline the importance of an individual assessment, while the presence of risk factors and exposure to traumatic experiences will not automatically lead to a disorder or adverse child outcomes.

##### Summary

Children’s vulnerabilities as a difficult temperament, mental health disorders, the impact of trauma, insecure and disorganized attachment, challenge the mentalizing capacity of the parent, and therefore represents a risk factor for the development of the parent-child relationship. Exposure to prenatal stress, anxiety, or depression, and as a possible consequence the development of a difficult temperament and behavioral problems in the child, can increase the risk of later psychopathology. Preventive approaches, such as stress reduction during pregnancy, psycho-education, and active treatment of the parent-child relationship will help parents when their offspring have long-term difficulties in self-regulation. Parental sensitivity, self-efficacy, and marital support and satisfaction are moderators that may attenuate the negative impact of fetal programming. In the presence of a mental disorder or disorganized attachment style in the child, parents need specific help in learning how to stay connected, during which they also self-regulate in a healthy manner.

#### Environmental Domain

The parent-child relationship develops within a larger ecological context with complex interactions between proximal factors (e.g., parenting) and distal factors (e.g., poverty) (Little et al., 2004). Research with a focus on social and economic determinants of mental health found evidence for a association between worse mental health and *poverty* (Silva et al., 2016; Lund and Cois, 2018), *low socio-economic status*, *low quality of neighborhood*, *housing problems*, *perceived discrimination*, *social isolation*, and *lack of social support* (Silva et al., 2016).

Barker et al. (2012) have found evidence for additional or independent impact of environmental risk factors (e.g., *low socio-economic status*, *low emotional, and practical support network*) along with parental mental disorders on children’s adverse outcomes. A meta-analysis by Cyr et al. (2010), shows an impact on child insecure and disorganized attachment by cumulative socio-economic risks of which low income, and *belonging to a minority group* is mentioned beside other risk factors. To explain this relationship between the accumulation of environmental risk factors and disorganized attachment, it is hypothesized that parents are so occupied with their personal and daily concerns, for example about money, housing, employment, that they withdraw from interacting with their child and are lacking in being predictably and safely available to them. Families with cumulative risk factors are at risk for chaotic living and child-rearing conditions, and neglect (Cyr et al., 2010).

A review of the effects of perinatal mental disorders on the fetus and the child (Stein et al., 2014) shows that children of parents with a mental disorder in low-income families are more affected by a parent’s mental disorder than children in more affluent families, and therefore interventions could be most important in such adverse circumstances.

Children of depressed mothers are exposed to significantly more risk factors than children of mothers without depression, with on average 2.3 risk factors for the former versus 1 risk factor for the latter (Barker et al., 2012). These risk factors include beside others, *low socio-economic status*, and *an inadequate emotional and practical support network.* Brockington et al. (2011) mentioned, as an example of the latter, families *without possibilities for alternative care* and Hosman et al. (2009) mentioned the *absence or low quality of adult and child professional care*.

All above mentioned authors argued, that social and economic risk factors should be targeted to improve (parental) mental health and reduce the number of risk factors to which children are exposed, especially risk factors that are responsive to intervention. In addition, the focus should be on enhancing protective factors such as social support, alternative care, and resources (Brockington et al., 2011; Falkov, 2012; Tharner et al., 2012).

##### Summary

Adverse socio-economic conditions and the presence of a (parental) mental disorder often occur simultaneously and reinforce each other. Children exposed to both are more affected than children exposed only to the latter. Consequently, besides treating the parental mental disorder, socio-economic risk factors should be targeted. Social support, for instance by the extended family, and possibilities for alternative care are important protective factors to take in account.

#### Summary of Intervention Targets

An overview of above mentioned risk factors, intervention targets, and the intended results which will act as a protective factor in the domain of concern is presented in Table 3.

**TABLE 3:** Risk factors and targets of intervention in different domains to prevent for intergenerational transmission of psychopathology and adverse outcome.

Domains	Parental disorder	Partner relationship and family life	Parent-infant relationship	Child	Environment
Risk factors	Early onsetSeverityComorbidityAddictionUnresolved (childhood) traumaAbsence of treatment	Early and single parenthoodAbsence of both parentsConflict or low quality in partner relationshipPsychopathology (addiction) of other parentChild abuse or neglect Unpredictable or lack of daily routines Imprisoning/criminal trouble family member Low level of education	Problematic parenting and parent-child relationship Disorganizing interactions between parent and infant	Difficult temperament Mental health disorders Early life stress Trauma/ACEsSignificant risk with ≥3 ACEsInsecure and disorganized attachment	Low socio-economic status Poverty/debts Housing problems Social isolation No supportive network Belonging to minority group Low quality of neighborhood Absence or low quality of support network, and professional care No possibilities for alternative care

Targets of Interventions	Treat the mental disorder Treat (childhood) trauma Treat addiction problems	Involve partner Address interparental violence and child abuse or neglect Enhance marital support and satisfaction Treat mental health problems of other parent Promote consistency in daily structure	Involve other parent Diminish problematic and disorganizing parental behavior Enhance parental mentalization and sensitivity Educate parent about child development Enhance parental efficacy	Treat infant problems, trauma and early life stress in the context of the parent-child relationship	Enhance social support (extended family, friendships) and if necessary, make provision for alternative care Reduce the impact of environmental risks (e.g., housing, financial, poverty, criminality, stress)

Results/protective factors	Better mental health	Warm and supportive marital and family life	Secure attachment	Optimal development	Increased capacity to carry out parental tasks Supportive network

Given the multiple interacting risk and protective factors and the large variety of family contexts, there is no universal approach for prevention and treatment. Little et al. (2004) argued that there are several points at which it is possible to intervene in the causal chain, and make a distinction between the role of proximal and distal processes in developmental deficiency, whereby the first operates nearby (e.g., poor parenting) and the latter far from the developmental deficiency (e.g., poverty). Children’s exposure to cumulative risks almost always has a greater impact on development than exposure to a single risk, and interventions targeting the full range of risks are more likely to be effective (Evans et al., 2013). There is also a suggestion of more benefit for children’s development when there is an accumulation of resources in the presence of risks (Evans et al., 2013). Therefore, intervention targets can differ and should be based on assessment of the individual profile of vulnerabilities and strengths to meet the needs of parent(s) and children in their contexts (Stein and Harold, 2015). A flexible, tailored for each individual family, resource-oriented intervention program, multi-faceted in addressing all modifiable risk factors and using different methods (individual, dyadic, group) seems to provide the best results (Nylen et al., 2006; Kersten-Alvarez et al., 2011; Evans et al., 2013; Schrank et al., 2015; Stein and Harold, 2015; Van Santvoort et al., 2015; Masten, 2018).

## Discussion

The aim of this article was to identify modifiable targets for intervention in the treatment of parents with serious mental disorders and their young children, and which targets should be prioritized to reduce the risk of transmission of psychopathology. The epidemiology of the intergenerational transmission of mental disorders provides grounds for concern about children of parents with a mental disorder, especially in infancy and early childhood, due to vulnerable periods in brain development and also a period of high sensitivity to stress (Agorastos et al., 2019). On an individual level, the probability is increased by cumulating risk factors and the absence of protective factors.

This paper provides a comprehensive review of intervention targets related to risk and protective factors that can help prevent the transmission of psychopathology from parents with young children in mental health care. An important conclusion of this review is that the literature shows that intervention targets are identified in different interacting domains, namely the parental, family, child, and environmental domains, as well as the developing parent-child relationship. A second conclusion is that given the multiple interacting risk and protective factors and the great variety of phenomenology of mental disorders and family and environmental contexts, there is no general approach for prevention and treatment to prevent parents and their young children from suffering intergenerational transmission of mental disorders (Schrank et al., 2015; Van Santvoort et al., 2015). Therefore, intervention targets can differ and should be determined by and based on individualized assessment of the risk profile to meet the needs of the parent(s), the child, and their relationships in their context (Nylen et al., 2006; Van Santvoort et al., 2015). A flexible, tailored, and resource-oriented program for treatment with different intervention methods (individual, dyadic, group) will promise the best results (Nylen et al., 2006; Schrank et al., 2015; Reupert and Maybery, 2016; Masten, 2018).

Unfortunately, this paper will not provide an answer to the question of which targets should be prioritized in treatment to reduce the risk of transmission of psychopathology. No research has been conducted that has analyzed which treatment targets have which impact in preventing intergenerational transmission or in reducing the risk factors associated with it (Christiansen et al., 2019). Despite this, some hypothesis can be made regarding urgency and aspects in treatment. In high risk samples where risk factors co-occur cascading models can guide the process of decision-making about which intervention target in which domain will have the greatest effect and therefore deserves priority. Cascading effects occur on processes that strengthen resilience as well as on processes that negatively reinforce each other resulting in a worsened situation.

Doty et al. (2017) present a cascading resilience model in which parenting interventions were postulated as an leverage point in promoting positive spillover effects in the domain of the parent (parenting efficacy, positive emotions, emotion regulation), the child (development, biological stress), the family (relationships, stress regulation), and functioning in the community (sociability, trust, social networks).

Harold and Sellers (2018) present in their review a cascade model in which interparental conflict is a feature of family stress which negatively affect children. They state that the target on interparental relationship is a direct source of influence on the parent-child relationship. Interparental support will have a moderating effect on the parental symptoms (see also McEwen, 2003), a positive effect on the quality of parenting and co-parenting (see also Korja et al., 2017; Hughes et al., 2019), security in the family, and will via this pathway improve child’s long-term mental health outcome. Therefore they argued that in the presence of interparental conflict, this should get a higher priority for intervention than interventions targeting the parent-child relationship. Considering the statements above, it will be obvious that the (co-)parent should be routinely involved during the assessment procedure (Stein et al., 2014).

Stepp et al. (2012) argued that mothers with a borderline personality disorder first need psychoeducation about childhood development and expectations, and skill training to promote consistency in warmth and parenting strategies, before they can benefit from attachment-based parent-child treatment.

This underscores the second conclusion that individualized assessment of the risk profile of the family should be made by professionals, before simply intervening on one domain, in order to decide which intervention targets will proceed the best spillover effects on other domains.

An important and often overlooked risk factor in mental health care is parents’ unresolved childhood trauma, which could play a significant role in causing their psychiatric symptoms (Allsopp et al., 2019). This is an important threat to parenting, because post-traumatic stress implies problematic self-regulation, which threatens the interpersonal regulation of the infant (Suardi et al., 2017). Hence, screening for unresolved childhood trauma during the assessment procedure and consideration of the impact on parenting and the parent-child relationship should be undertaken.

However, although treatment of the mental disorder and trauma of the parent is important, it will not automatically change undesirable patterns in the parent-infant relationship (Forman et al., 2007; Thanhäuser et al., 2017). Disruption to the parent-child relationship will affect both child and parent, with consequences for the future. In line with the transactional model (Sameroff, 2004), the child is at risk of behavioral and emotional problems, and in that case, parenting is more challenging and less satisfying. This poses a risk of worsening the parent’s symptoms, which could in turn further increase the child’s problems. Therefore, in mental health care practice, assessment of parenting and the parent-infant relationship should be an essential part of the overall assessment in the interest of both parent and child (Brockington et al., 2011; Falkov, 2012). As a consequence of their own problems, a parent may be biased regarding their own parenting behavior and their child’s behavior. Mothers with severe and pervasive mental disorders such as a personality disorder tend to view their struggles and behavior as ego syntonic, and Laulik et al. (2013) recommends for this reason that wherever possible, mental health care of these parents should include assessment of the attachment style of parent and child. Hence, assessment of the parent-infant relationship should be done through observation.

In addition to screening for parental trauma, the same should be done for the young child. It is important to assess the infant for traumatic experiences and exposure to parental stress from conception and treat them in the context of the attachment relationship (Chu and Lieberman, 2010).

Children are more affected by their parent’s mental disorder if their family is low in socio-economic status (Stein et al., 2014). Accumulation of environmental risk factors puts children at serious risk of developing a disorganized attachment style (Cyr et al., 2010), which is associated with later psychopathology (Sroufe et al., 2009). These risk factors make it challenging for parents to be consistently available and to regulate infants’ distress in a predictable and safe manner. Parents’ absorption in managing their daily troubles may result in chaotic living and child rearing conditions, and child neglect. Thus, contextual risk factors are important targets for intervention to diminish parental stress. In addition, it is important to enhance social support and search for possibilities for alternative care.

### Limitations

We have restricted our literature search mainly to reviews, which may have limited our identification of significant articles and potentially excluded other findings. Our choice to search for reviews was motivated by the broad field of science involved in our research question, with the possible risk that specific topics have been excluded simply because no review article about them has appeared. We have tried to address this risk by adding papers and meta-analytic studies and longitudinal research on missing issues. Another limitation is that the bulk of research focused on mothers. The influence of the psychopathology of fathers, as well as the possible buffering influence of a healthy father in case of a maternal mental disorder, has been less investigated. Furthermore, research into the transmission of parental psychopathology to offspring has mostly focused on a single mental disorder, and therefore does not offer guidance for practice in which comorbidity is present. The impact and interaction of other risk factors in transmission besides the parental mental disorder has seldom been investigated.

Despite these limitations, this review hopefully puts forward an useful overview of the present state of knowledge, identifying modifiable targets that are most helpful to parents in mental health care, enabling them to improve their parenting and develop a secure relationship with their young child, for the benefit of both. In addition, it will hopefully help professionals in adult and infant mental health care to help parents to break the cycle of intergenerational transmission of psychopathology.

### Implications for Clinical Practice

As shown in this review, risk factors in the transmission of psychopathology in different domains are highly interrelated and interactive, with negative cascade effects on both parents and children. Children of parents with a mental disorder are more likely to be exposed to more family and environmental risk factors than children whose parents do not have a mental disorder. Intervention targets and ports of entry for treatment are not simple to determine. A “one size fits all” intervention is not appropriate for parents with serious mental disorders and their young children. Thus, professionals need to carefully consider which intervention targets will be most likely to benefit each individual family. Specifically, which domains, which timing and combination of treatments, whether to focus on proximal or distal processes or on protective factors that moderate the influence of risk factors, the intensity of the intervention, and which professional(s) will be working with the family all need to be considered.

In practice, “no single service can fulfill the needs of both parent and child” (Falkov, 2012, p. 8), neither adult mental health care nor infant mental health care, so it is essential for these mental health care professionals to work together in close collaboration. However, adult mental health care and even child mental health care are both appropriate places to reach and help parents and their young children by assessing and treating the whole family including mental disorders, relational, and contextual problems. To address problems in the broader context of the family and society, a multi-agency approach including social services is needed.

## Author Contributions

All authors contributed to the design and method, and read and commented on the manuscript text of this review article. HS read all the included manuscripts of the review and put the manuscript into writing. All authors approved the final version of the manuscript.

## Conflict of Interest

The authors declare that the research was conducted in the absence of any commercial or financial relationships that could be construed as a potential conflict of interest.

## Publisher’s Note

All claims expressed in this article are solely those of the authors and do not necessarily represent those of their affiliated organizations, or those of the publisher, the editors and the reviewers. Any product that may be evaluated in this article, or claim that may be made by its manufacturer, is not guaranteed or endorsed by the publisher.
